# Astaxanthin Protects Against H_2_O_2_- and Doxorubicin-Induced Cardiotoxicity in H9c2 Rat Myocardial Cells

**DOI:** 10.3390/life14111409

**Published:** 2024-11-01

**Authors:** Roman Krestinin, Margarita Kobyakova, Yulia Baburina, Linda Sotnikova, Olga Krestinina

**Affiliations:** Institute of Theoretical and Experimental Biophysics, Russian Academy of Sciences, 142290 Pushchino, Russia; rkrestinin@bk.ru (R.K.); ritaaaaa49@gmail.com (M.K.); byul@rambler.ru (Y.B.); linda_sotnikova@mail.ru (L.S.)

**Keywords:** astaxanthin, H9c2 cells, cytotoxicity, doxorubicin, mitochondria, autophagy

## Abstract

Astaxanthin (AST) is a carotenoid that has positive effects on various organs and tissues. It also exhibits a cardioprotective action. In this study, the influence of AST on the survival of H9c2 cardiomyocytes under hydrogen peroxide (H_2_O_2_)- and doxorubicin (DOX)-induced cardiotoxicity was investigated. Under these conditions, the content of cytosolic Ca^2+^ was measured, and changes in the area of the mitochondrial mass, as well as in the content of the voltage-dependent anion channel 1 (VDAC1), the autophagy marker LC3A/B, and the pro-apoptotic transcription factor homologous protein (CHOP), were determined. It was found that AST removed the cytotoxic effect of H_2_O_2_ and DOX, while cell survival increased, and the mitochondrial mass did not differ from the control. At the same time, a decrease in the content of cytosolic Ca^2+^ and the restoration of the VDAC1 level to values close to the control were observed. The restoration of the CHOP level suggests a reduction in endoplasmic reticulum (ER) stress in cells. The results allow us to consider AST as a potential agent in the prevention and/or treatment of cardiac diseases associated with oxidative stress.

## 1. Introduction

Astaxanthin (3,3′-dihydroxy-β,β′-carotene-4,4′-dione, AST) belongs to a broad class of chemical compounds known as terpenes and is a xanthophyll because it has two extra oxygen atoms on each of its six-membered rings, differing from beta-carotene [1]. AST has beneficial properties, such as antioxidant, anti-aging, anti-inflammatory, antihypertensive and anti-cancer properties, which have expanded its use as a pharmaceutical, a cosmetic, a functional food, etc. [2,3]. In addition, AST has also been shown to have cardioprotective properties, and the AST diet is able to reduce the risk of cardiovascular disease [4].

For normal cell functioning, it is important to retain the structural and functional integrity of mitochondria, since mitochondria play an important role in energy metabolism, as well as in maintaining the redox state of cells and regulating the apoptosis. Mitochondria are one of the sources of ROS [5]. ROS play physiological and pathophysiological roles in biology and are closely related to redox signaling, but in some situations they can cause oxidative damage [6]. Mitochondrial dysfunction contributes to the development of oxidative stress, which can lead to various cell disorders and ultimately to cell death [7]. Recently, we have shown that AST improves the structure of cardiac tissue, as well as the functional state of rat heart mitochondria in isoproterenol-induced mitochondrial dysfunction [8,9].

It is known that the voltage-dependent anion channel 1 (VDAC1) is the protein localized in the outer membrane of mitochondria and plays a key role in mitochondrial function. VDAC1 mediates the fluxes of ions, nucleotides, and other metabolites across the outer mitochondrial membrane and controls metabolic and energetic crosstalk between mitochondria and the rest of the cell [10]. The role of VDAC1 in the pathogenesis of cardiac abnormalities is being studied intensively, and the results obtained indicate that VDAC1 may play a key role in cardiac pathology [11]. Moreover, it was reported that oxidative stress-induced injury of H9c2 myoblasts results in increased VDAC1 expression and oligomerization [12,13]. VDAC1 is involved in the transfer of Ca^2+^ from the endoplasmic reticulum (ER) to mitochondria [14]. In patients with hypertrophic cardiomyopathy, increased transcription levels of various genes including the VDAC1 gene were observed in septic tissue [15].

It is known that the endoplasmic/sarcoplasmic reticulum (ER/SR) is involved in the accumulation of Ca^2+^. A decrease in Ca^2+^ content in the cell can result in the disruption of the SR lumen, which ultimately leads to ER stress [16,17]. It is believed that the long-term ER stress activates a signaling pathway that induces transcription factors associated with apoptosis. One such factor is the C/EBP homologous protein (CHOP), which is activated in the PERK-ATF4-CHOP signaling pathway [18].

The goal of the present study was to assess the protective effects of AST against H_2_O_2_- and doxorubicin (DOX)-induced cardiotoxicity in H9c2 rat cardiomyocytes. H_2_O_2_ and DOX are potent oxidants that promote oxidative stress and cause oxidative damage to cardiomyocytes [19,20]. Therefore, these compounds are used to induce cytotoxic effects in healthy cells. We investigated the effect of AST on the mitochondrial mass, the cytosolic Ca^2+^ content, and the content of VDAC1 and CHOP while the cytotoxic effect of H_2_O_2_ and DOX were present. The results showed that AST attenuated H_2_O_2_- and DOX-induced cardiotoxicity in H9c2 cardiomyocytes, suggesting its potential use in the prevention and treatment of cardiovascular diseases.

## 2. Materials and Methods

### 2.1. Cell Culture and Treatment

The H9c2 cell line, derived from the rat myocardium, was purchased from the American Type Culture Collection (ATCC, Rockville, MD, USA). Cells were cultured in Dulbecco’s modified Eagle’s medium (DMEM, Thermo Fisher Scientific, Waltham, MA, USA) supplemented with 10% fetal bovine serum (FBS, Thermo Fisher Scientific, Waltham, MA, USA), 100 U/mL penicillin, and 100 μg/mL streptomycin (Sigma, St. Louis, MO, USA) in a humidified incubator with 95% air and 5% CO_2_ at 37 °C. To induce cardiotoxicity in cardiomyocytes, H_2_O_2_ (Sigma, Louis, MO, USA) was used at different concentrations: 0, 25, 50, and 100 μmol/L. The rate of the inhibition of cell proliferation was 50%, depending on the concentration in subsequent experiments. H9c2 cells (2.5 × 10^3^ cells/well) seeded in a 96-well plate were treated with H_2_O_2_ (0–100 μM) or DOX (0–100 μM) for 24 h, or cells were treated with AST (5, 10, 15, 20 μM) for 4 and 6 h.

### 2.2. Cell Viability Analysis

Cell viability was assessed using the resazurin recovery method. Cells at a density of 2.5 × 10^3^ cells/well were seeded in a 96-well plate. After 24 h, the cells were treated with AST (Macklin Inc., Shanghai, China) (5–20 µM), H_2_O_2_ (0–100 μM), and DOX (0–100 μM). Twenty four hours after the addition of the test substances, resazurin (Sigma-Aldrich, USA) was added to each well at a final concentration of 100 µg/mL, and cells were incubated for 4 h under CO_2_ incubator conditions. Fluorescence analysis was performed on an Infinite F200 microplate reader (Tecan, Männedorf, Switzerland) at an excitation wavelength of 535 nm and an emission wavelength of 595 nm. The data are given as the percentage of the control (untreated cells). The number of dead cells after incubation with AST (10 μM), H_2_O_2_ (100 μM), and DOX (100 μM) was determined using a commercial Cytotoxicity Detection Kit (LDH) (Roche Diagnostics GmbH Waldhof, Mannheim, Germany) according to the manufacturer’s recommendations. Optical density was measured at 490 nm using an iMARK plate reader (Bio-Rad, Hercules, CA, USA). Cells treated with 1% Triton X-100 (Helicon, Moscow, Russia) were used as a control. The data are presented as percentage.

### 2.3. Confocal Microscopy

For confocal microscopy experiments, H9c2 cells were seeded in 35 mm Petri dishes (15,000 cells/cm^2^) and treated with 10 μM AST, 100 μM H_2_O_2_, or 100 μM DOX for 1 h. In a combined study of AST and H_2_O_2_ or AST and Dox, pre-incubation with AST was performed for 4 and 6 h, respectively. After the incubation, the cells were washed three times with 2 mL of HBSS and incubated in 2 mL of HBSS supplemented with 1 μg/mL Bisbenzimide Hoechst 33342 (H33342, Sigma-Aldrich, St. Louis, MO, USA) and 150 nM MitoTracker Green FM (Cell Signaling, Danvers, MA, USA) at 37 °C for 30 min in a thermostat without CO_2_. After staining, the cells were washed 3 times with HBSS buffer. Fluorescent images of cells were obtained using a fluorescence scanning confocal microscope Leica TCS SP-5 DM6000 CS (Leica Microsystems, Wetzlar, Germany).

### 2.4. Analysis of Cytosolic Ca^2+^ Content

The cytosolic Ca^2+^ content was changed using the fluorescent dye Fluo-4 AM (Sigma-Aldrich, USA). To do this, cells were seeded onto a 96-well plate at a concentration of 1 × 10^6^ cells/well and incubated with the test drugs for 24 h. Next, the cells were pelleted at 250× *g* for 5 min and t = 25 °C, washed once in PBS, and a cell suspension of 1 × 10^6^ cells/mL was prepared in PBS. Cells were stained by adding 2 μM Fluo-4 AM to the suspension and were subsequently incubated for 30 min in a CO_2_ incubator. After staining, cells were washed once in PBS. The change in cytosolic Ca^2+^ content was carried out on an Infinite F200 PRO (Tecan, Männedorf, Switzerland) at an excitation wavelength of 494 nm and an emission wavelength of 516 nm.

### 2.5. Western Blot Assay

H9c2 cells were incubated with AST (1000 µM) and H_2_O_2_. After 24 h, cells were washed twice with ice-cold PBS and centrifuged at 1500× *g* for 3 min at room temperature. The precipitate was dissolved in lysis buffer with the addition of protein kinase/phosphatase inhibitors. After rotating the samples at 4 °C for two hours, the samples were centrifuged at 13,000× *g* for 10 min. The resulting supernatants were measured for protein concentrations by the Bradford method and dissolved in Laemmli sample buffer (Bio-Rad, Hercules, CA, USA), heated to 95 °C for 5 min. The resulting lysates (20 μg per lane) were separated by 12.5% SDS-PAGE. Proteins were then transferred from the gel to a nitrocellulose membrane (0.2 μm) using Western blotting (Bio-Rad. Hercules, CA, USA). The membrane was blocked in Roti-block solution (Carl Roth GmbH + Co., Karlsruhe, Germany) at room temperature for one hour. The membrane was then incubated with primary antibodies as described in the instructions. Antibodies to VDAC1 (Abcam, Cambridge, UK), LC3A/B (Cell signaling, Danvers, MA, USA), and CHOP (FineTest, Wuhan, China) were used. GAPDH (Santa Cruz, Dallas, TX, USA) was used as a loading control.

### 2.6. Statistical Analysis

Statistical analysis was performed using one-way ANOVA and appropriate post hoc analysis (Student–Newman–Keuls). Differences were considered significant at *p* < 0.05.

## 3. Results

### 3.1. The Cytotoxic Effect of AST, H_2_O_2_, and DOX on the Survival of H9c2 Cardiomyocytes

The possible protective effect of AST was identified using models in which the damage to and the death of H9c2 rat cardiomyocytes were achieved using cytotoxicity inducers, such as H_2_O_2_ [21] and DOX [22]. Previously, we observed that, when incubating cells with different concentrations of AST together with H_2_O_2_ or DOX in the range from 5 to 100 μM, only 5 and 10 μM AST showed a protective effect (Supplementary Figure S1).

The combined effect of AST, H_2_O_2_, and DOX on the viability of H9c2 cells was assessed for one, four, and six hours of incubation, since the change in cell viability depended not only on the concentration of the used substances but also on the time of incubation with AST.

**Figure 1:**
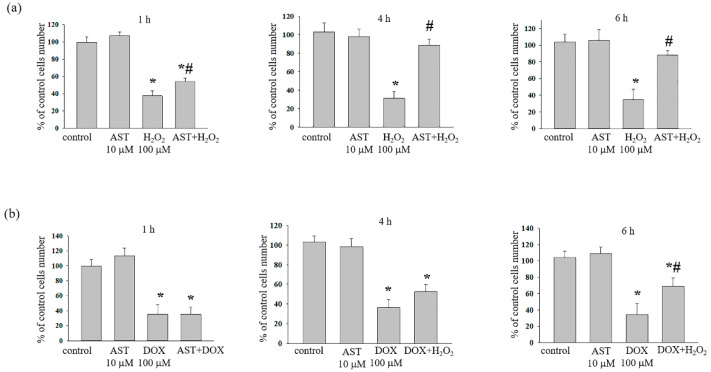
Dependence of the viability of H9c2 cells on the concentration of AST (10 µM), H_2_O_2_ (100 µM), and DOX (100 µM). (**a**) Cytotoxic effect of AST, H_2_O_2_, and AST+H_2_O_2_; (**b**) cytotoxic effect of AST, DOX, and AST+DOX. The proportion of living cells was determined one, four, and six hours after the addition of substances using the resazurin reduction method. The number of living cells in the intact culture (control, without drug treatment) was taken as 100%. Data are presented as the mean ± standard deviation (n = 10). * p < 0.05 indicates significant changes compared to the control (intact culture); *# p* < 0.05 indicates significant change compared to the corresponding values of H_2_O_2_ or DOX.

Here, we show the combined effect of 10 μM AST with 100 μM H_2_O_2_ or 100 μM DOX (Figure 1). Cardiomyocytes in an amount of 2.5 × 10^3^ were incubated with AST for 1, 4, and 6 h, after which cytotoxicity inducers were added and cell viability was measured. When incubated with AST for 1, 4, and 6 h, cell viability was not different from the control. After the addition of H_2_O_2_ (100 μM) to cells, the viability decreased by 66.6 ± 5% relative to the control.

The combined effect of AST and H_2_O_2_ (1 h incubation with AST) decreased cell viability by 50% compared to the control and increased it by 37% relative to the effect of H_2_O_2_ alone. Four-hour incubation with AST followed by the addition of H_2_O_2_ resulted in an increase in cell viability by 2.5 times compared to the effect of H_2_O_2_ alone and did not differ from the control.

The incubation of cells with AST, regardless of the incubation time, did not reveal a significant effect; the viability did not differ from the control. Although cell viability was reduced by 65% with H_2_O_2_ (100 μM), the incubation of cells with 10 μM AST for 4 h followed by the addition of H_2_O_2_ (100 μM) was not different from the control. After six hours of incubation with AST, the addition of 100 μM H_2_O_2_ resulted in a 10% reduction in cell viability.

Figure 1b shows a change in cell viability after incubation with AST for six hours followed by the addition of DOX. As can be seen, after one, four, and six hours of incubation with AST, cell viability did not differ from the control. The addition of DOX to the cells decreased the viability by approximately 65–70% compared to the control. The addition of DOX to AST-incubated cells resulted in a decrease in cell viability by 65% (after 1 h incubation with AST), 50% (after 4 h incubation with AST) and 30% (after 6 h incubation with AST) compared to the control.

Thus, the protective effect of AST was more significant at concentrations of 10 μM AST with 100 μM H_2_O_2_ when cells were incubated with AST for four hours and 10 μM AST with 100 μM DOX when cells were incubated with AST for six hours.

**Figure 2:**
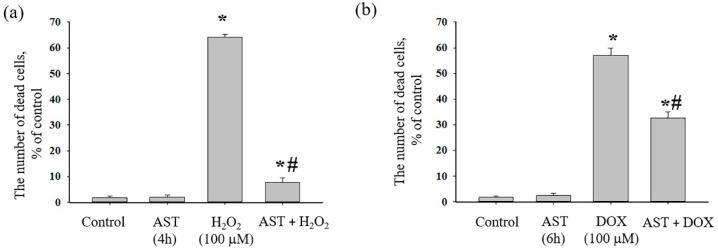
Effect of AST and its combined action with H_2_O_2_ and DOX on the death of H9c2 cardiomyocytes. (**a**) Cell death in control (untreated cells), with AST, H_2_O_2_, and AST+H_2_O_2_; (**b**) cell death in control (untreated cells), with AST, DOX, and AST+DOX. Cells treated with 1% Triton X-100 were considered 100% dead and were taken as a positive control. Data are presented as the mean ± standard deviation (n = 8). * p < 0.05 indicates significant change compared to the control (intact culture); *# p* < 0.05 indicates significant changes compared to the corresponding values of H_2_O_2_ or DOX.

Next, we investigated the effect of AST and its combined action with cytotoxicity inducers on the survival/death of H9c2 cells (Figure 2). Triton X-100 (1%) was considered a control and implied 100% cell death.

Figure 2a shows changes in the number of dead cells in the presence of AST, H_2_O_2_, and the combined effect of AST and H_2_O_2_. It is seen that the number of dead cells in the control (untreated cells) did not exceed approximately 2%. When cells were incubated for 4 h with 10 μM AST, the number of dead cells, as in the control, did not exceed 2%, whereas in the presence of H_2_O_2_ (100 μM), the number of dead cells reached 65%. With the combined effect of AST and H_2_O_2_, the number of dead cells did not exceed 7%. Figure 2b shows changes in the number of dead cells in the presence of AST and DOX, as well as the combined effect of AST and DOX. In the control (untreated cells) and during the 6 h incubation of cells with 10 μM AST, the number of dead cells did not exceed approximately 2%. In the presence of DOX (100 μM), the number of dead cells reached 57%. The combined action of AST and DOX resulted in a decrease in the number of dead cells relative to the action of DOX alone and was approximately 32%.

**Figure 3:**
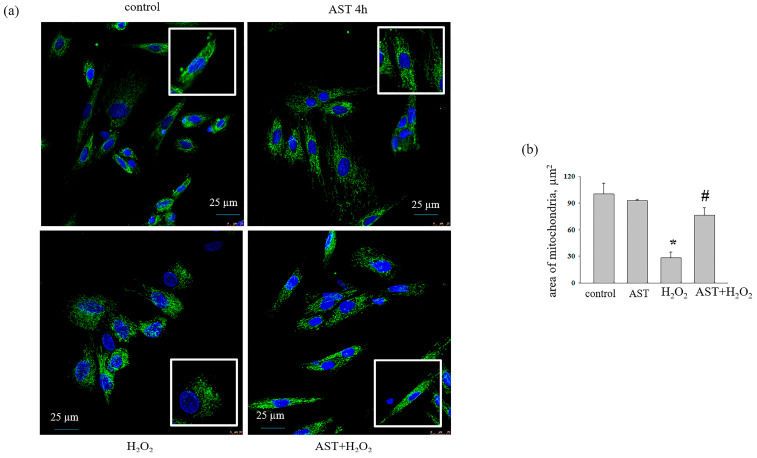
Effect of AST (10 μM) and H_2_O_2_ (100 μM) on the area of mitochondrial mass in H9c2 cells. (**a**) Mitochondria stained with MitoTracker Green FM; the cell nuclei stained with Hoechst 33342; (**b**) mitochondrial area calculated using the ImageJ software. The data are presented as the mean ± standard deviation (n = 5). * p < 0.05 indicates significant change compared to the control (intact culture); *# p* < 0.05 indicates significant changes compared to the corresponding values of H_2_O_2_.

**Figure 4:**
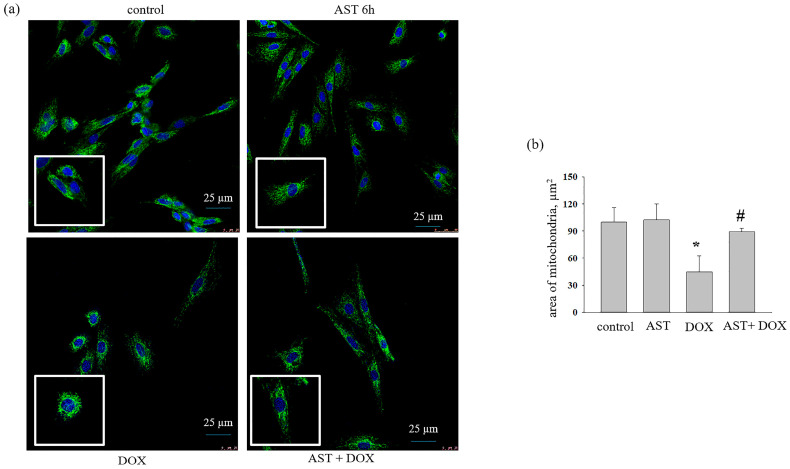
Effect of AST (10 μM) and DOX (100 μM) on the area of mitochondrial mass in H9c2 cells. (**a**) Mitochondria stained with MitoTracker Green FM; cell nuclei stained with Hoechst 33342; (**b**) the mitochondrial area calculated using the software ImageJ. Data are presented as the mean ± standard deviation (n = 5). * p < 0.05 indicates a significant change compared to the control (intact culture); *# p* < 0.05 indicates significant changes compared to the corresponding values of DOX.

Next, we studied the effect of AST (10 μM), H_2_O_2_ (100 μM), and DOX (100 μM) on the morphological characteristics of mitochondria in a monolayer of H9c2 cardiomyocytes. We noticed that, after the addition of H_2_O_2_ (Figure 3) or DOX (Figure 4) to the cells, mitochondria concentrated around the nucleus in the cell, and their numbers decreased compared to the control. Therefore, we measured the area of mitochondria in a monolayer of cells under our experimental conditions. To do this, an equal number of cells was checked, and the area of mitochondria in each cell was calculated using the ImageJ software (https://imagej.nih.gov/ij/ (accessed on 12 June 2024)).

### 3.2. The Effect of AST, H_2_O_2_, and DOX on the Change in Mitochondrial Mass in H9c2 Cardiomyocytes

Figure 3a depicts the data of confocal fluorescent microscopy where the mitochondrial mass is stained with MitoTracker Green FM, and cell nuclei are stained blue (Hoechst 33342) in the presence of AST, H_2_O_2_, and of AST in combination with H_2_O_2._ Figure 3b shows changes in the area of mitochondria in the cell. Incubation of cells with AST for 4 h did not change the mitochondrial mass, while hydrogen peroxide significantly reduced it by 70%. When AST and H_2_O_2_ were used together, the mitochondrial area did not differ from the control. With doxorubicin used as an inducer of cytotoxicity, almost similar results were obtained (Figure 4).

Figure 4a shows the data for confocal microscopy, and Figure 4b represents the area of mitochondria in cells. The incubation with AST for 6 h did not change the area of mitochondria relative to the control. The addition of DOX decreased the mitochondrial mass by 50%. With the combined action of AST and DOX, the mitochondrial area approached the control values.

### 3.3. Effect of AST, H_2_O_2_, and DOX on the Content of Cytosolic Ca^2+^ in H9c2 Cardiomyocytes

It is known that Ca^2+^ is an important secondary messenger, which is involved in many cellular processes, such as protein synthesis and secretion, gene expression, cell cycle progression, metabolism, apoptosis, and others [23].

**Figure 5:**
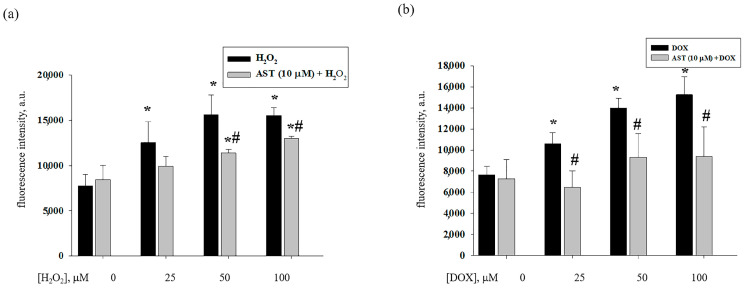
Effect of AST on the content of cytosolic Ca^2+^ in H9c2 cardiomyocytes during H_2_O_2_- and DOX-induced cytotoxicity. (**a**) the content of cytosolic Ca^2+^ in the presence of AST and H_2_O_2_; (b) the content of cytosolic Ca^2+^ in the presence of AST and DOX. Fluorescence intensity of intact cells was used as a control (without drug treatment), standardized to 2.5 × 10^5^ cells. Data are presented as the mean fluorescence intensity ± standard deviation (n = 6). * p <0.05 significant changes compared to the corresponding control, *# p* <0.05 significant change compared to H_2_O_2_ or DOX.

Therefore, at the next stage, the effect of AST on the level of cytosolic Ca^2+^ in H9c2 cardiomyocytes was studied while cytotoxicity caused by H_2_O_2_ and DOX was experienced (Figure 5). Figure 5a,b shows changes in the content of cytosolic Ca^2+^ under the combined influence of AST with H_2_O_2_ and AST with DOX based on the changes in fluorescence intensity with the use of Fluo 4 fluorescent dye. As seen from Figure 5a, the content of cytosolic Ca^2+^ increased at a concentration of H_2_O_2_ of 25 μM by 40% and at concentrations of 50 and 100 μM by two times compared to the control. The incubation of cells with 10 μM AST for 4 h followed by the addition of H_2_O_2_ reduced the content of cytosolic Ca^2+^ by 20% compared to 25 μM H_2_O_2_, by 35% compared to 50 μM H_2_O_2_, and by 20% with 100 μM H_2_O_2_.

With DOX used as an inducer of cell cytotoxicity, similar results were obtained. The content of cytosolic Ca^2+^ increased in the range of 25–100 µM DOX from 35 to 90%, respectively (Figure 5b). When cells were treated with 10 μM AST, the content of cytosolic Ca^2+^ decreased by 40% compared to 25 μM DOX, by 35% compared to 50 μM DOX, and by 40% compared to 100 μM DOX. Thus, AST reduced the content of cytosolic Ca^2+^ in H9c2 cardiomyocytes during H_2_O_2_- and DOX-induced cytotoxicity. At the same time, it exhibited a protective effect.

### 3.4. Effect of AST, H_2_O_2_, and DOX on the Level of VDAC1 and CHOP in H9c2 Cardiomyocytes

**Figure 6:**
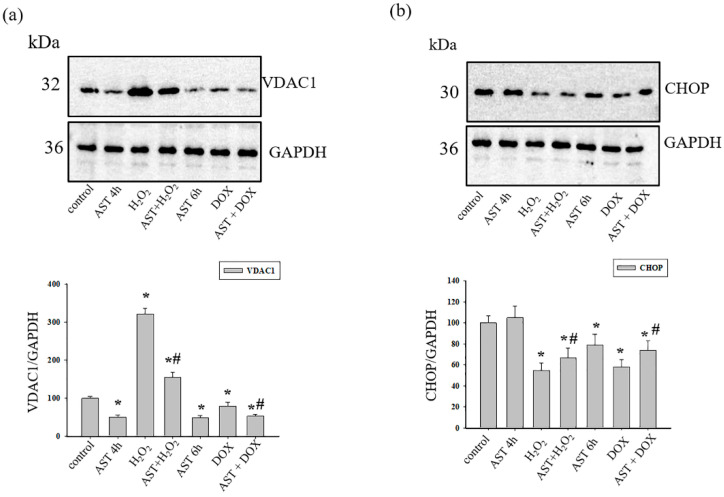
Effect of AST on the content of VDAC1 and CHOP in H9c2 cardiomyocytes during H_2_O_2_ and DOX-induced cytotoxicity. (**a**) Upper part: Western blot stained by VDAC1 antibody; low part: graphs quantifying changes in the protein content normalized to GAPDH; (**b**)—upper part: Western blot stained by CHOP antibody; low part: graphs quantifying changes in the protein content normalized to GAPDH. Protein levels in cell lysate (without additives) served as control (100%). Data are presented as the mean ± standard deviation (n = 4), * *p* < 0.05 indicates significant changes compared to the control. # *p* < 0.05 indicates significant changes compared to H_2_O_2_ or DOX.

It is known that there are three isoforms of the voltage dependent anion channel (VDAC); however, VDAC1 is the most abundant isoform in mitochondria. It has been shown that VDAC1 levels can increase under conditions of oxidative stress. At the next stage, we examined changes in the content of VDAC1 and CHOP under our experimental conditions (Figure 6).

Figure 6 shows Western blot stained with antibodies to VDAC1 and CHOP. The antibody to GAPDH was used as a loading control. AST (4 h) reduced the level of VDAC1 by 50%, while H_2_O_2_ increased it almost three times compared to the control (Figure 6a). With the combined effect of AST and H_2_O_2_, the content of VDAC1 increased by 1.5 times compared to the control but decreased by 1.5 times compared to the action of H_2_O_2_ alone. The content of VDAC1 in cells incubated with AST for 6 h decreased by 50% relative to the control. The addition of DOX to cells reduced the level of VDAC1 by 20% relative to the control, but the combined effect of AST and DOX led to a decrease in the level of VDAC1 by 45% in comparison with the control. On the other hand, DOX increased the content of VDAC1 by 40% relative to the effect of AST (6 h) alone; however, the combined effect of AST and DOX resulted in a decrease in the VDAC1 level compared to the effect of DOX alone.

Figure 6b (upper part) shows Western blot stained with the antibody to CHOP, lower part represents quantitative changes in immunostaining normalized to GAPDH. A 4 h incubation with AST did not change the content of CHOP, while H_2_O_2_ reduced it by 45% compared to the control. When AST and H_2_O_2_ were used together, the level of CHOP decreased by 30% relative to the control but increased by 15% relative to the effect of H_2_O_2_ alone. A 6 h incubation with AST reduced the content of CHOP by 20% and with DOX by 40% compared to the control. The combined effect of AST and DOX reduced the level of CHOP by 25% relative to the control but increased it by 15% relative to the effect of DOX alone.

## 4. Discussion

The growing incidence of cardiovascular diseases in developing countries is one of the most pressing health problems today. Myocardial infarction is the most common form of coronary heart disease and a cause of premature death [24]. It is known that mitochondria are involved in the etiology of various diseases, such as neurodegenerative and cardiovascular diseases, diabetes, various forms of liver and musculoskeletal diseases, sepsis, and psychiatric disorders [25]. Moreover, oxidative stress and the deterioration of Ca^2+^ homeostasis are considered important factors in mitochondrial dysfunctions, as they can lead to cell death [26]. Among effective substances that can be used as protection against mitochondrial dysfunction is the dietary antioxidant astaxanthin. In the present study, we studied the effect of AST on the viability of H9c2 cardiomyocytes after the addition of cytotoxicity inducers such as H_2_O_2_ and DOX. We studied changes in the mitochondrial mass, the cytosolic Ca^2+^ content, as well as in the content of VDAC1 and CHOP under these conditions. Analyzing the effect of different AST concentrations on cell survival, we came to the conclusion that lower AST concentrations of 5 and 10 μM with the addition of high concentrations of cytotoxicity inducers (H_2_O_2_ and DOX) were more effective (Supplementary File S1). In the present study, 10 μM AST was selected as the most suitable for the protective effect of AST. At this concentration of AST, cell viability increased despite the cytotoxic effects of H_2_O_2_ and DOX. The protective properties of AST were more pronounced in H_2_O_2_-induced cytotoxicity than in DOX-induced cytotoxicity, probably because the mechanisms of action of H_2_O_2_ and DOX on cells differ. The mechanism of the cytotoxic action of DOX is mediated not only by oxidative stress, as in the case of H_2_O_2_, but it is also associated with the suppression of DNA synthesis and translation due to DNA intercalation, as well as the formation of double-strand breaks due to the inhibition of DNA topoisomerase II activity [27]. Interestingly, AST has the opposite effect on cancer cells. It has been shown that astaxanthin can suppress cancer proliferation in human non-small cell lung cancer, bronchioloalveolar carcinoma (A549), and squamous cell carcinoma (H1703) cells by reducing the colony formation ability of the cells at a concentration of 20 μM [28]. AST enhanced tumor cell sensitization to chemotherapy and suppressed its side effects. In this context, AST can be considered as a potential candidate for use in combination therapy in the fight against cancer in patients with developing resistance to traditional therapy [29]. The opposing effects of AST in healthy and cancer cells suggest the presence of different targets and mechanisms of its action.

It is believed that the balance between the rate of mitochondrial biogenesis and degradation may determine the number of mitochondria in any cell [30]. Here, the H9c2 cell line was grown under different experimental conditions. Mitochondria were tagged with MitoTracker Green FM and quantified using the ImageJ software (https://imagej.nih.gov/ij/ (accessed on 12 June 2024)). Our results demonstrate that incubation of cells with AST for 4 and 6 h did not change the number of mitochondria, while the addition of H_2_O_2_ and DOX led to a significant decrease in the number of mitochondria compared to the control. Moreover, we noticed that mitochondria under the conditions of cytotoxic action of H_2_O_2_ and DOX were shorter than in the control and during the incubation of cells with AST. This indicates the initiation of mitochondrial fragmentation with the addition of cytotoxicity inducers. Cun-dong Fan et al. showed that AST blocked a homocysteine-induced drop in the mitochondrial membrane potential (ΔΨm) and mitochondrial fragmentation in H9c2 cells [31]. We previously showed that H_2_O_2_ and DOX decreased the mitochondrial potential (ΔΨm) [32], while AST restored it. Recently, we examined changes in the expression of prohibitin (PHB) under similar conditions and found that the protein content was reduced in the presence of H_2_O_2_ and DOX [32]. It is known that PHB depletion severely affects mitochondrial morphology in *C. elegans* body wall muscle cells [31]. In normal muscle cells, mitochondria appeared tubular, elongated, and well structured. With PHB loss, mitochondria showed up fragmented and disorganized [31]. Similarly, decreased PHB expression resulted in the accumulation of fragmented mitochondria in MEF and HeLa cells [33,34]. According to our results, it is logical to assume that in the presence of cytotoxicity inducers, a decrease in the PHB content could lead to a reduction in mitochondrial mass and their fragmentation. Increasing levels of PHB resulted in restoration of mitochondrial mass to the control values.

VDAC1, located on the outer mitochondrial membrane, is known to be an important protein for mitochondrial function. It mediates the transport of ions, nucleotides, and other metabolites across the outer mitochondrial membrane and the energetic crosstalk between mitochondria and the cell and is also involved in the release of apoptogenic proteins that initiate apoptotic cell death [10,30,35]. It has been shown that VDAC1 expression levels were increased and oligomerization occurred in H9c2 myoblasts under oxidative stress [12,13]. Moreover, VDAC1 was found to be involved in the detrimental Ca^2+^ transfer from the endoplasmic reticulum (ER) to mitochondria [14]. VDAC1 overexpression is associated with myocardial abnormalities in common pathological conditions. Other researchers have shown that increased levels of VDAC1 expression in a rat model of cardiac hypertrophy were induced by renal artery ligation [36]. According to our studies, H_2_O_2_ and DOX caused a cytotoxic effect in H9c2 cardiomyocytes and increased the content of cytosolic Ca^2+^; at the same time, the overexpression of VDAC1 was observed in cells. However, incubation with AST led to a decrease in Ca^2+^ in the cytosol and a decrease in the level of VDAC1 expression. The more pronounced expression of VDAC1 during H_2_O_2_-induced cytotoxicity may be explained as occurring through mechanisms different from those of DOX.

The endoplasmic/sarcoplasmic reticulum (ER/SR) is an intracellular organelle involved in Ca^2+^ accumulation and is thought to be sensitive to changes in Ca^2+^ homeostasis. Depletion of Ca^2+^ from the lumen of the SR can impair its function, which can result in stress of the ER. ER stress results in organelle damage and dysfunction, leading to apoptosis [16,17]. In addition, Bcl-2 family proteins, together with the transcription factor CHOP, are involved in the regulation that mediates ER stress-induced apoptosis [16]. Moreover, it was shown that, in neonatal rat cardiomyocytes, upon initiation of oxidative stress by H_2_O_2_, there was a decrease in CHOP expression [37]. In our experiments, we observed that, with decreased cell viability in the presence of H_2_O_2_ and DOX, the level of CHOP decreased, while AST increased the viability of cardiomyocytes and increased CHOP expression. There is an interorganellar crosstalk between the ER and mitochondria, which plays a crucial role in various pathological processes, especially in Ca^2+^ homeostasis [38]. VDAC1 is involved in Ca^2+^ transport from the ER to mitochondria, while the ER (the major intracellular Ca^2+^ storage system) can transmit Ca^2+^ signals to mitochondria [39]. Multiple proteins are involved in the linkage of the ER and mitochondria. It has been reported that the ER inositol 1,4,5-triphosphate receptor (IP3R) associates with VDAC1 in the outer mitochondrial membrane, forming a pathway for Ca^2+^ transport [40], while under ER stress conditions, CHOP activates the IP3R-mediated release of endoplasmic reticulum Ca^2+^ into the cytosol [41,42]. It should be noted that, in ischemic disease of the heart, apoptosis of cardiomyocytes mechanically associated with ER stress and mitochondrial dysfunction can lead to the occurrence of myocardial infarction [43,44]. Reduced Ca^2+^ signaling may serve as a possible mechanism to prevent ER stress-mediated mitochondrial Ca^2+^ overload and mitochondrial membrane permeability impairment, ultimately protecting against the initiation of cell death. This section of the study requires further study in more detail.

## 5. Conclusions

As a result of the cytotoxic effect of H_2_O_2_ and DOX, cell survival decreased, while an increase in the cytosolic Ca^2+^ content and a dramatic increase in the expression of VDAC1 were observed. It should be noted that the number of mitochondria under these conditions decreased and they were fragmented. The mitochondria of cardiomyocytes were more sensitive to Ca^2+^, as indicated by the effects of its increase in the cytosol. At the same time, the content of the transcription factor CHOP decreased. We have previously found that under these conditions, a decrease in the membrane potential of mitochondria occurs. Damaged mitochondria with low potential accumulated in cells and were not removed by autophagy. The pre-incubation of cells with AST, despite the addition of cytotoxicity inducers to the cells, resulted in a decrease in the cytosolic Ca^2+^ content, and mitochondria were more resistant to Ca^2+^. This resulted in the restoration of VDAC1 levels. In addition, the number of mitochondria did not differ from the control. AST effectively improved cell survival and reduced the cytosolic Ca^2+^ overload, possibly through the disruption of the CHOP-VDAC1 signaling pathway, thereby protecting mitochondrial morphology. According to our findings, we suggest that AST involves the mitochondrial pathway as a signaling pathway, through which AST exerts a protective effect on cardiomyocytes.

## Data Availability

The data presented in this study are contained within this article.

## Supplementary Materials

The following are available online at https://www.mdpi.com/article/10.3390/life14111409/s1. Figure S1: The dependence of the viability of H9c2 cardiomyocyte cells on the concentration of AST, H_2_O_2_, DOX and on the time of incubation with AST.
