# Supplementary material for: Astaxanthin Protects Against H2O2- and Doxorubicin-Induced Cardiotoxicity in H9c2 Rat Myocardial Cells
Source: Life (Basel). 2024 Nov 1;14(11):1409. doi: 10.3390/life14111409 (PMC11595901; doi:10.3390/life14111409)
Supplement: Supplementary file 1 [file life-14-01409-s001.zip › life-3243839-supplementary.pdf]

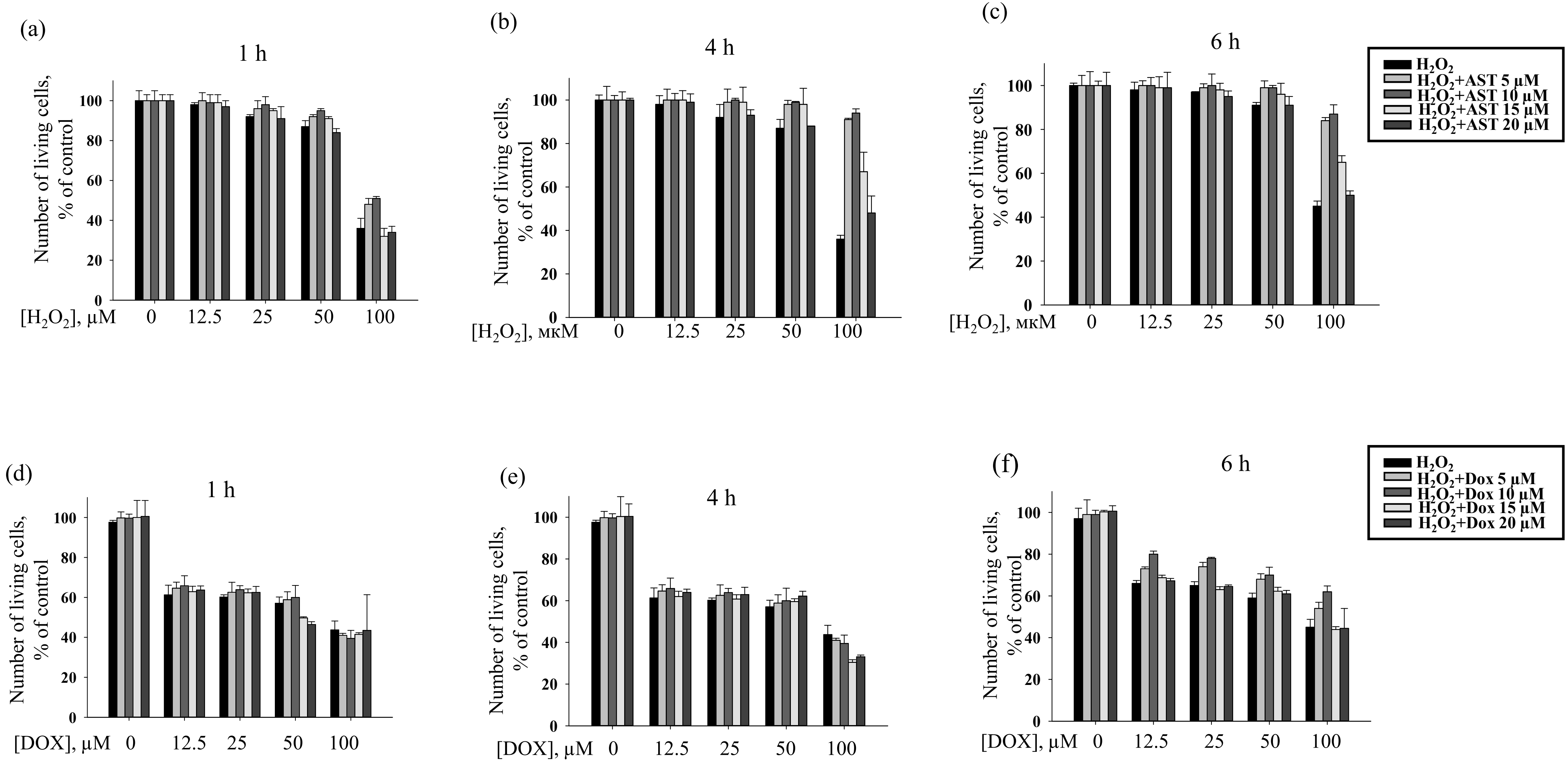

Figure S1. The dependence of the viability of H9c2 cardiomyocyte cells on the concentration of AST, H_2O_2, DOX and on the time of incubation with AST. The cells were incubated with AST for one (a, d), four (b, e), and six (c, f) hours. The data are presented as the mean ± S.D. from six separate experiments. The control consisted of untreated cells.
